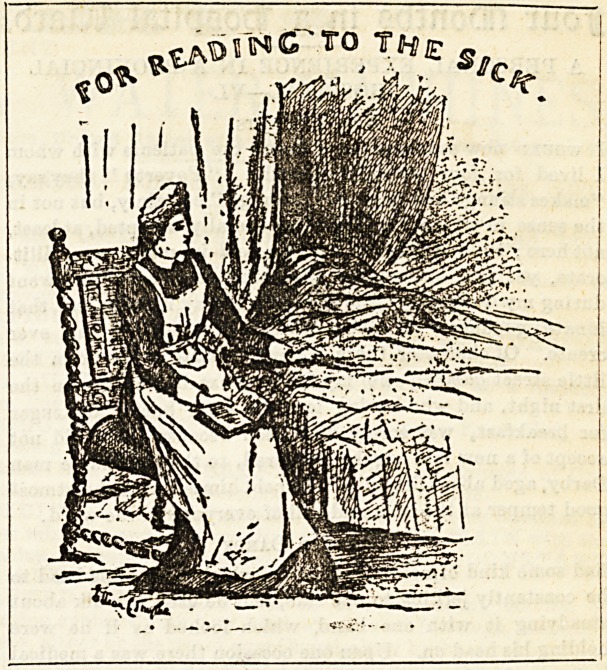# The Hospital Nursing Supplement

**Published:** 1892-06-25

**Authors:** 


### "The Hospital" Nursing Mirror.

Being the Extra Nursing Supplement of "The Hospital" Newspaper.

Contributions for this Supplement should be addressed to the Editor, The Hospital, 140, Strand, London, W.C., and should have the word "Nursing" plainly written in left-hand top corner of the envelope.

### En Passant.

CHILDREN.—At Miss Bowditch's Cottage Home, to which such substantial additional accommodation has been recently built, children from the Invalid Aid Association have been received, and most satisfactorily nursed during the last five years. In 1890 the Association expended £120 in sending children there, and last year they spent £150 in the same way.

HOLIDAYS.—Some of our nurse friends are off to Ireland and some are going abroad, and others are meditating walking tours, while others are taking their holiday at home. Two nurses write to us from the Brassey Home, telling of picnics, breakfasts on the beach, and other joys too numerous to mention, which they have had during their stay there. "There are no restrictions," writes one nurse, "and one feels so at home." This is what a home should be, there is nothing so unpleasant as to be hemmed in with rules. We have had many letters asking advice about holidays and we shall be very glad to make any more suggestions.

THE Q.V.J.N.I. IN DUBLIN.—A very well attended meeting met at the Archbishop of Dublin's House at Dublin last week for the purpose of assisting in the permanent establishment of a training home for Catholic district nurses in the city in connection with the Queen Victoria Institute of Nursing. Last year Lady O'Hagan visited Dublin, and at the request of the Central Council of the Institute, opened a home at 21, Mary Street. The good which the very small staff has been able to effect in the short space of ten months has encouraged the promoters to make an effort to extend the ministrations of the nurses to the sick poor over a wider area than was possible before.

CLAPHAM MATERNITY HOSPITAL. — The third annual report of this little hospital is quite a cheering piece of literature, for it tells of a total of 2,200 cases successfully treated, and of only one death, and that was a poor woman who succumbed to pneumonia in her own home. As a correspondent of the *British Medical Journal* very justly says: "A mortality of one mother in 2,200 maternity cases is a record which could scarcely be beaten." We are proud of this proof of the care and skill of lady doctors having achieved so brilliant a success, and especially are we glad to hear of figures which unequivocally contradict those audacious statistics recently offered for the bewilderment of the Select Committee.

PRIVATE NURSES AND THEIR WAYS. — There is not very much to be gained by a correspondence on this question—we wish that the only desirable solution could be arrived at, namely, a little give and take on both sides. There are houses to which private nurses go where disagreeables are many, and we know that under such circumstances duty becomes difficult. A nurse need not remain at a house where she cannot obtain ordinary necessaries, but such a state of things as this is not often met with. Let a nurse's chief thought be how much she can do to relieve her patient and how little trouble she need cause the household; but, on the other hand, nurses are mortal, and the strain of their work demands thought and care on the part of their employer as regards rest and nourishment. If the injunction, "be courteous," were better obeyed in its fullest sense, it would save much trouble between employer and employed.

THE SUMMER NUMBER OF THE "GRAPHIC."—Our readers who have not already seen it, may like to know that in the summer number of the *Graphic*, just published, appears a monograph by Miss Honnor Morten, entitled "The Story of a Nurse." It is illustrated by Miss Mary Gow, and the subjects for the pictures are both well chosen and executed. This number of the *Graphic* would be a welcome addition to nurses' libraries, and those who have spare copies might remember this fact and send them to the hospitals, where they would be welcomed.

BRABAZON HOME OF COMFORT.—This home was founded by the Countess of Meath, then Lady Brabazon, for the reception of members of the Girls' Friendly Society, who, not being eligible for hospitals, would probably linger out their days in a workhouse infirmary. Invalids are also admitted who require medical care and nursing to give them back lost strength. Nine free beds are taken by different dioceses, and they elect their own cases. An immense deal of good is done by patiently nursing back to health girls whose usefulness as workers was well nigh lost. Four industrial girls are taken in the Brabazon Home and are trained for service, and with two or three exceptions, they have all turned out well. The Secretary would gladly welcome a few more subscribers of 5s. to the Home; in fact anything from a few pence upwards will be gratefully received by Miss Cazenove, Ravenleigh, Betchworth.

WOLVERHAMPTON QUEEN VICTORIA NURSING INSTITUTION.—We are glad to hear of the steady progress after three years' work of this institution. The asssociation is now affiliated with the Q.V.J.I.N., and receives a grant of £40 a year for the district nurses' fund. A second nurse is now at work in the Blakenhall district, and Nurse Reader, the first nurse to the sick poor in St. Paul's district, is now a "Queen's Nurse." There are now thirty on the staff, namely, twenty-two trained nurses, two district nurses, and six probationers in training, and the year's report on the efficiency and conduct of the nurses is excellent. When the nurses are established in a house of their own, there will be little to be desired to add to their prosperity. We hope to hear that pensions for the nurses are made a part of the scheme of this institution. Some more financial support given to the district nursing fund will, we hope, be forthcoming soon.

POISONS AS INFANTILE NOURISHMENT.—What a grandly original path to fame was discovered last week by the coroner's jury who decided at Chatham that a certain child died "from natural causes, viz., bronchitis," in spite of the doctor's evidence that morphia-poisoning was the primary cause of death! A cough mixture supplied by a herbalist had been administered, and the presence of the poison in it, as well as the symptoms of the sufferer, were not questioned. It is almost as poetic a verdict as the one given at Belfast the other day on a similar occasion, but the dose in the latter case was half a teaspoonful of laudanum, which the mother had given on her own responsibility, and we find that "poisoning by inadvertence" is the recorded decision in this case. By whatever names these Irish or English intelligences choose to disguise these transactions, it seems about time that a stop should be put to the habit of treating poor babies to a dietary of poisonous drugs, even though such a practice does not always end in manslaughter.

### Ventilation, Disinfection, and Diet.

By P. Caldwell Smith, M.D.

### XI.—DISINFECTION—(*concluded*).

In Measles—Typhus—Whooping Cough—Erysipelas—Diphtheria—Tubercular Diseases—Typhoid—Cholera.

With measles the same precautions do not require to be taken. The infective poison of the disease is probably more virulent at the commencement of the disease, and isolation at that time until convalescence is the principal prophylactic means at our disposal. Before sending the child back to school the clothing, bed, &c., should be thoroughly disinfected.

With regard to typhus fever, it has in the first place to be remembered that this is a disease of over-crowding and poverty, and to prevent it this must be remedied. The disease is one that both doctors and nurses require to exercise great caution in, as it is very liable to affect those in attendance. To prevent infection from occurring the typhus patient should have a very large amount of cubic space, and the most free ventilation is absolutely necessary. Care should be taken that the breath of the patient is not inhaled, and all excreta should be received into a disinfectant solution.

After convalescence, generally three weeks from the outset of the disease, the room, clothing, bed, and bedding ought to be disinfected in the usual way. No case of typhus should be treated at home, but it should at once be removed to a fever hospital.

Whooping cough is a very infectious disease, and a very fatal one in children, but little has been done in the way of prevention. The infection in this disease is principally existent in the expectoration, and this should be destroyed at once by burning. Pieces of lint should be used for receiving it, and these should be immediately burnt. The child should of course be isolated till the characteristic whoop has disappeared completely. The clothing, linen, and room should then be disinfected as usual.

Erysipelas should not be treated in general hospitals, but in special wards in fever hospitals. Care should be taken that no one suffering from a wound is brought into contact with it, and the nurse attending on a patient with this disease should not on any account be allowed to undertake midwifery cases for at least three weeks, and she, as well as her clothing, should be carefully disinfected. All dressings, bandages, &c., used in treatment of a case should, when taken off, be burned at once.

In diphtheria, all discharges from the throat and nose should be received on lint and burnt at once. The patient must be isolated, and care should be taken by attendants not to inhale the breath of one suffering from this disease. It has been recommended that all those in attendance on diphtheritic cases should have their throat sprayed with a 1 per cent. solution of carbolic acid three times a day. The patient should not be allowed to mix with others for fourteen days after all the local symptoms have disappeared. The usual methods of disinfection should be used for clothes, rooms, and bedding.

A very great deal might be said about tubercular disease in its various forms, but the means we are more directly called on to use are for the prevention of its spread from patients. It is necessary, therefore, that all sputa from a consumptive should be received into a vessel, half filled with a 10 per cent. solution of creolin or carbolic acid, and that this, when full, should either be buried or burnt, preferably the latter. The best way to do this is to pour it at the back of a good red fire, ensuring in this way complete destruction. All linen, bedding, &c., should be disinfected and cleaned separate from the other household linen, &c.

If a patient suffers from turbercular disease of the bowels then the excreta have to be disinfected. In fact this might be done in all cases, as the patient suffering from consumption of the lungs frequently swallows some of the bacilli, and these, of course, pass off by the bowels.

I have already said something regarding disinfection in typhoid fever, but owing to its importance, and the frequency with which you will be called to nurse the disease, I shall reiterate and emphasise these former remarks. The infection exists only in the bowel discharges, and these should be carefully disinfected. I prefer the solution of creolin, while others pin their faith to a one or two per cent. solution of corrosive sublimate. The excreta should be allowed to remain in contact with the disinfectant before being thrown into the soil-pipe. The ideal form of disinfection for these would be to receive them into sawdust impregnated with some disinfectant and then burned. As also stated before, absolute cleanliness and disinfection of the hands is important, more especially before sitting down to meals. The linen, bed, and bedding should be disinfected as before. Any towels or linen which cannot thus be dealt with should be soaked in solution of creolin and then boiled. This is quite as good as disinfection by steam.

In villages and country houses where no drainage system exists, the excreta should be disinfected as above, and then buried eighteen inches in the earth.

In cholera the same process has to be gone through, but if possible greater precautions should be taken. The bacillus exists in the stools, but in this disease these are so frequent and copious that the bed linen, &c., are very liable to be soiled. Greater care has, therefore, to be taken in the disinfection of these at once. A strong solution of creolin or carbolic acid may be used, the linen, &c., remaining in contact with this for twenty-four hours. They may then be washed and dried. Everything in contact with a cholera patient should be disinfected, and no food should be taken by the nurse in the sick room. Before doing so she should go into another room and disinfect her hands thoroughly, first taking off her nursing dress, which in this disease should be a special one, from the liability of it being soiled by the frequent discharges, it is safer also to disinfect the vomited matters.

It is a very safe plan never to take food directly with the hands, but always with a fork or spoon. Absolutely the same precautions should be taken in all cases of severe diarrhœa during a cholera epidemic, as these very frequently cause spread of the disease before being recognised as Asiatic cholera. The room should after convalescence be disinfected in the usual way. If any go abroad, to India, &c., where this disease is very common, I would advise you to keep these facts in your minds, as I am perfectly sure they will go a long way to protect you from taking the disease. Of course I have not entered into the question of drinking polluted water or milk, as that is beyond the province of this lecture.

### Where to Go.

On Thursday, June 30th, a musical and dramatic performance in aid of the Royal Hospital for Diseases of the Chest will take place at the Lyric Theatre at three p.m. Prices of seats range from one guinea to one shilling, and an excellent programme is promised.

The Countess of Aberdeen will preside at the annual meeting of the Invalid Children's Aid Association at four p.m., on June 30th, at 20, Hanover Square. A large and interesting gathering of friends of sick children is anticipated, and speeches will be made by the Hon. Sir Charles Freemantle, Canon Fleming, Dr. Broadbent, Mr. Timothy Holmes, and others.

A matinée, at half-past two, will be given at the Strand Theatre on June 28th, in aid of the Children's Hospital, Paddington Green, when "Lady Browne's Diary" and "The Gavotte" will be given. Miss Minnie Bell, Miss Silvia Grey, Miss Rosina Brandram, and other well-known names will, it is hoped, secure a large audience. Tickets from 10s. 6d. to 1s.

### Nursing of the Sick English Soldier in India.

*To the Editor of* The Hospital.

Sir,—We all owe a debt of intense gratitude to H.R.H. the Duke of Connaught for his evidence before the Wantage Committee. The dying-off of the young soldier and young officer is sad beyond conception. We lose some 400 young officers and boys every year of this deadly fever, and numbers who do recover are worthless ever again. What is wanted is to draw English attention to our nursing system, which is most defective. We have no Medical Staff Corps, that is to say, trained soldier nurses, to work in the hospitals. In half-a-dozen stations we have three or four English Sisters, but they are made to do the actual nursing as well, without any trained help under them. As doctors, we have over and over again asked for trained orderlies to do the executive nursing, but cannot get them. I beg you will publish the annexed letter, which was published locally in India, as it shows what is needed. The manly evidence of the Duke of Connaught, full of sympathy with the soldier, has endeared him to the whole of those exiled Englishmen who are serving the Indian Government in India. Long life and prosperity to him!—Yours faithfully,

1892. Medical Officer.

### THE CRYING WANT IN INDIAN ARMY HOSPITALS.

An Indian Medical Staff Corps.

An open letter to the Hon'ble Lieut.-Genl. Brackenbury, R.A., Member of the Council of the Viceroy of India.

Sir,—I beg to address you in this open letter, as it is not possible for any official communication from me to reach you. From the time of your appointment as Member of Council I have built much on the good work you could do for the Army hospitals in India, as I am aware of your large experience under the Red Cross in 1870-71, and have read your valauble reports on the same sent on to the National Aid Society in England.

The crying want in the British Hospitals in India is a corps of trained European or Eurasian soldier orderlies to nurse the soldier in severe illnesses. You are aware that there is in India a corps, "save the mark!" called the "Army Hospital Native Corps," made up of underfed, underpaid, undrilled, and undisciplined native waifs and strays. These men do the fatigue work needed in this climate within the hospitals. In some of the larger hospitals there are nursing Sisters, ladies from England who are in many cases entirely overworked from having to carry out executive nursing duties over many bad cases, and they are thus overworked because they have no reliable European or Eurasian soldier orderlies to whom to entrust any detail nursing work with the sick. The Sisters do not work in the front of the army, nor in epidemics of cholera, and their services are limited to a few of the larger Indian garrisons; but men are sick and dying in every garrison, and receive only rough-and-ready nursing.

That nursing is given by ordinary soldiers taken by chance from the sick man's company, and, being ignorant of the work, and returned to duty after the recovery or death of their patient, are in no sense to be considered as trained nurses in typhoid fevers. In the old easy-going, long-service days of the past, it was possible to get old soldiers in the battalions who made very fair nurses. Now all the men are younger, less experienced, and are being constantly needed for musketry, military training, and other duties. The loss to the sick is excessive.

We need, above all things, a permanent body of trained men to be given to us, who would draw the extra pay as nursing orderlies, and who would be promoted to non-commissioned rank in an Indian Medical Staff Corps on the lines of the English Medical Staff Corps. Eurasian, or country-born lads would find in such a corps a fair field for work and eventual promotion into the warrant grades of the subordinate medical service, after training in a special course at colleges. In asking for these men, it is quite impossible to hide from you that very grave consequences are occurring from the introduction of the three or four nursing Sisters to do the nursing work in our larger hospitals. Short-handed as they are, for the purpose of grasping their work, a most dangerous and entirely objectionable system of concentrating serious and typhoid cases is now going on. That is to say, for the purpose of obtaining good nursing, numbers of typhoid and serious cases are crowded into the Sisters' ward, and a perpetual stream of such men are being sent, with hardly a break, into the same rooms, until the places become saturated with a typhoid atmosphere. The advantages of the Sisters' nursing are, therefore, being daily handicapped by the fatal system of concentration of fevers going on in the hospitals.

The feeling of fear on the part of the sick, and the actual objection on the part of the doctors to submit their cases to concentration, is being everywhere felt, and one may hear urgent requests from the sick not to be sent into the Sisters' wards. Relapses of cases from remaining week after week in a continually poisoned fever ward are not uncommon, and the depression produced on the sick, by seeing day by day their comrades die, has also to be considered. No one would acknowledge the need of trained orderlies as their helpmates more than the Sisters themselves, who are often "dead beat" from trying to accomplish an impossible task, viz., nursing twenty or thirty typhoid cases at one time. Inspections of hospitals are made in the cold season, but visit a typhoid ward on a July day, and see a Sister who has been on duty for twelve or fourteen hours over a ward full of sick. It is a monstrous overtask, and it results entirely from the want of a reliable corps of trained orderlies, or the placing on the unattached list of a body of trusted men chosen specially to care for the sick.

It is a terrible thought that this English soldier, whom Mr. Stanhope obtains with such difficulty in England, is so little cared for when sick in India, as to have only a worthless native corps in the hospitals, and only chance comrades to care for him as nurses.

If a trained and permanent European or Eurasian corps was formed, you could diminish two very expensive bodies now employed—viz., the apothecary class and the nursing Sister—as the sergeants of the orderlies could be trained as compounders as in England, and could care for the more unimportant cases in certain wards in every hospital, reducing the apothecary class by one-fourth or so.

I can assure you the matter is urgent for peace, but for war it is all important. There the Sisters will be far away from the front, and the chance comrades will be quite unattainable, and only the wounded and the doctors will remain. I beg you will think of this matter now in peace, and as you are unbiassed, and with large experience, will give it a fair investigation. The matter is certain to attract attention in England in the near future, and it is well to look into every abuse that could check recruiting or interfere with re-engagement. A soldier in prison is watched and cared for by a paid detached private soldier warder; why should he, when ill, have to trust to chance help and unpaid and ever changing attendance.

I was personally assured by a medical officer of very high rank, so high, indeed, that his view could affect a large section of the army, that he disapproved entirely of concentration of typhoid fever; but I have never heard of any order being issued in his sphere of influence to check concentration. The officer when sick is never so crowded up or concentrated with other typhoid cases, nor is there any special need for the private man to be so treated; quite the reverse. He needs air, space, and quietness.

I feel certain that medical officers, apothecaries, and nursing Sisters agree that the present system of ever-changing orderlies is shockingly bad and unjust in every way to the sick. While many ask for a permanent European contingent of orderlies, it is possible that the legitimate claims of our Eurasian fellow citizens could be met by organising a Eurasian Medical Staff Corps to do this special nursing work. They would serve under the orders of the medical warrant officers, and be protected in the discharge of their work. They would receive the rank and pay as in the English Medical Staff Corps, and be graded as privates, corporals. and sergeants, with extra duty pay for special work done, Speaking English and Hindustani, not liable as a rule to typhoid fever, and capable of intelligently carrying out orders, and they would be a god-send in war and peace, and would go far to satisfy the reiterated demand of the medical officers for better help in their work. Besides acting as nursing orderlies, the Eurasian orderlies would also do for writers, clerks to P.M.O.'s, and storekeepers in hospitals, in all of which details of work the medical service is very incomplete, and entirely unready for the strain of real war.—I am, sir, &c., INDIAN MEDICAL STAFF CORPS.

### Everybody's Opinion.

### THE NEEDLESS DREAD OF NURSING SMALL-POX CASES.

"Veritas" writes: I have read with some surprise the remarks of "J. M." in your issue of the 11th inst. anent the loathsome character of small-pox; for certainly, however well it may be nursed, a confluent case is one of the most loathsome objects one can behold. "J. M." believes it would be found that the chances of taking small-pox if nursed on enlightened lines are not greater than in any other infection; there I must differ from her, for it is a well-known fact that small-pox has a stronger infecting power than any other disease, but persons may with the utmost safety nurse this disease who have been successfully and recently revaccinated, and surely no nurse is ever allowed to undertake small-pox work unless she has been revaccinated. Unfortunately the nature and value of vaccination are so little considered, if at all, in a nurse's training, that we need not be surprised at nurses shrinking from small-pox. I think that all nursing institutions should make it a rule for every nurse to be revaccinated when she joins their staff. "J. M." further states that the dread of nursing small-pox belongs to many years back, but again I cannot agree with her; for, not long ago a case was admitted into the hospital over which I preside, and both the nurses who took the duty felt a certain amount of dread, and were several days before they could feel confident that they were in no personal danger. These were both nurses of considerable general and fever experience, and sensible, strong-minded women, and were afterwards very thankful to have overcome their fear, and anxious for further experience, as I was myself when I had once had a taste of small-pox work.

### WANT OF SYMPATHY.

"Nurse M." writes: In common with many others I am surprised at the remarks made by the "Superintendent of the South Australian Hospital for the Insane," when he says, "I am not in the habit of expressing sympathy but reprimanding his attendants for not being on the alert when they are attacked by a patient." I dare say he is well-guarded when he goes his rounds, so that his great "alertness" is not necessary. It is such unkindly expressions that make asylum attendants incline to be discourteous to those in authority over them. No attendant who has read The Hospital for this week will, I am sure, regret being so many miles from kindly Dr. Cleland, and none but what will praise and think well of the editor for the way he has defended the poor attendants on the insane.

### A HOLIDAY IN SWITZERLAND.

"Nurse in Yorkshire" writes: After a great deal of enquiry as to expense, route, &c., we find we shall be able to take the following places in our tour (we leave London on August 1st and return August 30th) we go to Rotterdam, Amsterdam, Cologne, steamer to Bingen, Heidelberg, Basle, Berne, Lausanne, steamer to Geneva, from there steamer to Bouveret at the bottom of the lake, then on to the St. Gothard, Goschenen, Andermatt, Lucerne Basle, and home to London. The cost will be £15 for the month inclusive of everything except stimulants. No luggage must be brought excepting a change of linen and dress in a locked bag, which each nurse must be responsible for. The travelling will be second-class by rail, and first-class by steamer, and any further information can be obtained through the Editor of The Hospital, who has very kindly helped us in our holiday plan. Any more nurses wishing to join must please send name and references as soon as possible.

### Notes on Novelties.

Silveret.—We think the Silveret plate powder, manufactured at 95, Great Saffron Hill, E.C., by the Nubian Manufacturing Company, will commend itself to nurses from the fact of its lessening and shortening the labour of cleaning silver and electro-plate, and its seems equally good for tins and such-like articles. It is very easy to use and makes no mess, and removes stains in a wonderfully rapid fashion.

Underclothing.—We have received such excellent example of ladies' clothing from the Artistic Underwear Company that we are able to speak in the highest terms of their productions. The company supply their goods direct to their customers at first cost, and are therefore in the position of offering a very superior article at popular prices. The materials employed are of far better quality than we have hitherto met with for the same prices. They are well finished and prettily trimmed, and the long cloth employed contains no dressing. Theillustrated catalogue is to be had at the company's depôt, 33, Fore Street, E.C.

### Appointments.

Coldstream Hospital.—Miss Jessie Calder, presently Matron in the Stephen Hospital, Dufftown, Banffshire, has been elected Matron to the Coldstream Hospital, out of upwards of 50 applications.

Mary Hewitson Memorial Cottage Hospital, Keswick.—Miss Grace Dodgson, at present a nurse in the Cumberland Infirmary, Carlisle, has been appointed Nurse Matron of the Mary Hewitson Memorial Cottage Hospital, Keswick, out of seventy applicants. She was trained at the Wirral Children's Hospital, Birkenhead, also at the Cumberland Infirmary.

### Presentation.

District Nurse, Shipley.—A correspondent writes: Miss Annie Lumb, the Church District Nurse for Shipley and Saltaire, near Bradford, was on Friday, June 10th, presented with a gold bracelet, suitably inscribed, as a token of the gratitude in which her services are held. It was subscribed for only by those who had been her patients, who, however, were of all denominations. The presentation was made by the Vicar of the Parish, the Rev. Mr. Cribb. This is a proof of the real necessity of such trained aid for poor people, and is particularly gratifying at this period of commercial depression.

### Notes and Queries.

### Queries.

Can some of my fellow-workers supply me with information about Jersey? I very much wish to go there for my annual holiday, but know nothing about its advantages or disadvantages for that purpose, and seeing all the correspondence about places for nurses' holidays, I hope some one can tell me a little as to the best route to take from London, also a few particulars about expenses, &c.

Could any body tell me of a home or institution where an aged woman (73) could be received. She is suffering from harmless delusions, the after effect of influenza, and could pay 8s. to 10s. a week.—*P. A. M.*

### Answers.

*N. C. Bath.*—A letter addressed to you from us has been returned through the dead letter office. There will be a vacancy at Bellot's Charity, Bean Street, Bath, in about a fortnight, for which you could apply.

*Sairey Gamp.*—We will answer your first query next week. Get Mrs. Dacre Craven's "Guide to District Nurses," price 2s. 6d., at MacMillan's.

*L.M.P.*—Write to the Dean, Mason's College, Birmingham, and ask him if he will admit you. Only urgent replies can be given a written answer.

*A. F.*—Your communication was too late for last week's issue. We had already gone to press. We shall enquire into the matter as soon as possible, and see where blame lies, but certainly could not think of inserting your letter until we actually had seen such a state of affairs. Individually you could one and all help to raise the tone and meanwhile have patience, and do your best to your patients.

*Country Matron.*—Certainly not, it would be almost impossible for such a rule to be carried out if it existed anywhere, and we are quite sure it does not.

*E. M. R.*—Write to Miss Maud Lobb, Uplands, Loughton, Essex; they have just moved from Lambourne. Their new home is close to Epping Forest, not far from Loughton Station. Children of the poorer classes are taken at 7s. 6d. a week.

### Nursing Homes.

### VII.—THE ROYAL PORTSMOUTH, PORTSEA, AND GOSPORT HOSPITAL.

FIVE years ago the nursing arrangements at this institution left much to be desired. The year previously, Miss Tillett, who had been trained at the Glasgow Western Infirmary, was appointed Matron, and, thanks to her initiative, the whole of the nursing arrangements have been re-organised, and are now in many respects excellent.

Although there is no separate nurses' home, a portion of the old buildings has been set apart for the accommodation of the nurses, and each nurse has her own bedroom, all of these apartments being airy and comfortably furnished. In a few instances the rooms contain two beds, but, with one exception where this is the case, screens are provided, which necessarily add to the comfort of the nurses. Some of the old wards have been converted into cubicles, and now constitute the very best types of this class of bedroom which we have yet met with. The nurses' dining-room is a fine apartment, comfortably furnished, with pictures and a fair supply of books. In addition to this there is a lecture-room, containing a piano, where entertainments are given, and the nurses are allowed to amuse themselves in any way they think best.

In addition to the ordinary nursing staff, there are ten private nurses for private families, which are in much request. The charges are one guinea a week for ordinary cases, £1 10s. for night nursing, and £1 1ls. 6d. for infectious diseases. A fee of 3s. 6d. is charged for a single day, and 5s. for a single night. The charge for attending on surgical operations varies from 5s. to 21s., according to the case. The travelling expenses of each nurse, and an allowance of 2s. 6d. a week for washing, are also charged to the patients.

Probationers are trained at Portsmouth, the age being from 22 to 36. During the first year they are supplied with board, lodging, and washing, but each probationer must provide her own indoor dress of the hospital uniform pattern. All probationers are charged an entrance fee of one guinea, and are paid £18 wages for the second year, and £20 for the third year, with board, lodging, washing, and uniform in addition. At the end of each year a bonus of £6 is given on the recommendation of the Matron. At the end of the third year the wages are again increased. At the expiration of the first month's trial the probationer is required to sign an agreement, which provides that she will serve the Royal Portsmouth Hospital for three years, including the first year's training, and if she desires to be freed from this engagement earlier, she undertakes to pay £3 by way of forfeit money. A limited number of special probationers are received on payment of 30 guineas for the year, but special arrangements are sometimes made for a shorter period, on payment of 25 guineas for six months, or 13 guineas for three months' residence in the hospital. All payments are made in advance, and no money is returned in the event of a special probationer leaving for any cause. They are provided with board, lodging, and laundress. We may add that there is an excellent tennis lawn for the nurses in the gardens attached to the hospital.

The Committee have expressed their high sense of the work done by the Matron, Miss Tillett, especially in regard to the private nursing establishment. This establishment produced an income in 1891 of £365, whilst the training fees paid by nurses yielded under £92, *i.e.*, altogether £457. On the other side, the salaries of the nursing staff amounted to rather less than £430, so that the receipts from nurses' earnings and fees more than sufficed to pay the wages of the whole nursing staff of the hospital. In this connection we would press upon the Committee the justice of their undertaking to pay half the premiums of the members of their nursing staff, many of whom are members of the Royal National Pension Fund for Nurses. We have no doubt that their attention has only to be directed to this point to ensure its early adoption.

### COMING.

IT happens frequently to some of us that our illness becomes so prolonged, that the hope of a restoration to health grows more distant day by day, till we almost sink into despair and wonder how we shall bear the "life in death" which seems to be our portion. But there is hope even for us; our Lord whispers in tenderest accents "Fear not little flock, for it is your Father's good pleasure to give you the Kingdom." A kingdom not of this world, the glory and beauty of which must pass away, but the kingdom, the glorious Salem, the heavenly city, where are the mansions Christ has prepared for His beloved ones, and to which He Himself will come and take us.

It is a very awful subject this "coming of our Lord," and one to which we should not naturally have turned for comfort and consolation, and yet from studying the various passages of Holy Scripture which speak of it there is no doubt that our Christ meant us to find both peace and joy in looking forward to His appearing. Sinners as we are, we can find our springs of comfort here. Though we are full of faults and shortcomings and failures, we may, nay, we ought to rejoice in this blessed prospect.

In order to be prepared for His coming our Lord tells us to "watch." The time of our sojourning here must be spent in patient waiting for the coming of the bridegroom; our loins must be girded, our lamps burning lest we should not be ready for the summons, for the Master will come in an hour when we least expect Him, at even, or at midnight, or at the cock-crowing, or in the morning; we must watch therefore lest He find us sleeping.

As we lie still, pondering on our past life, do we not see the emptiness of the pleasures which this world offers us, do we not realise that our earthly treasures are a burden which moth and rust corrupt, and which thieves break through and steal, while, if we truly love God, however insufficiently, and fix a bright faith on His mercies and promises, our joy and treasures will be growing more and more in that sure storehouse, where neither rust nor moth can corrupt nor thieves break through and steal. Our hearts are necessarily fixed on that place where our true riches abide and thankfully contemplate the prospect of the better country in the land which now seems very far off, but which we should be all looking for and hasting unto. "To see Christ is bliss, to know Him life, to love Him happiness, to possess Him, heaven."

> Weep not, for unto you is given,
> To watch for the coming of His feet,
> Who is the glory of our blessed heaven;
> The work and watching will be very sweet
> Even in an earthly home,
> And in such an hour as ye think not, He will come

### Four Months in a Hospital Ward.

### A PERSONAL EXPERIENCE IN A PROVINCIAL HOSPITAL—VI.

### Our Patients.

I would now say something about the patients with whom I lived for four eventful months. "Poverty" they say, "makes strange bed fellows," "strange" certainly, but not in the sense in which the adage is generally accepted, at least, not here; rude of speech, may be, and in many cases illiterate, yet not one among the many that came and went during my long stay who did not display more or less that innate goodness of heart which no education can ever create. Of character there was an infinite study, from the little street *gamin*, who lay in the next bed to me on the first night, and who having lent me some butter and sugar for breakfast, was sorely distressed because I would not accept of a new laid egg he proffered, to that estimable man Darby, aged about fifty-five, who held himself with the utmost good temper at the beck and call of everyone in the ward.

### Poor Old Darby had some kind of nervous affection which caused his head to be constantly jerking to one side, and he used to walk about steadying it with one hand, which looked as if he were holding his head on. Upon one occasion there was a medical lecture to be given to members of the profession, out somewhere in the town, and Darby was chosen as a subject. It was an event of great importance in the eyes of us patients, and we felt that for the honour of the medical ward our representative should not be wanting, at least in the matter of dress. Now poor Darby's wardrobe was sadly the worse for wear and deficient in many important details, such as collar and necktie, and the like; so one lent him these items, another a coat and waistcoat, another a red cotton pocket handkerchief that might have passed for silk in the gas light, and these, with a borrowed hat and umbrella, and a pair of black woollen gloves, so completely transformed our nodding Mandarin that his own wife, had he called upon her, would not have known him. The ward maid crowned our handiwork by placing a rose in his buttonhole, and with a visible swelling of his "manly bosom" our medical subject went forth to his tryst.

### A Wreck of an Irishman.

Another patient was Martin, also a general favourite, the wreck of a fine, tall Irishman, about thirty-eight years of age, broken down by the constant wheeling of too heavy barrow-loads of coal from a canal-boat to a furnace. Although but a labourer, he was a highly intelligent man, taking great interest in his newspaper, and had good sound opinions on many subjects. On the Irish Question he was particularly eloquent, and would discourse by the hour on the rights and wrongs of his unhappy countrymen. He had the irrepressible spirits of his race, and was never at loss for an anecdote to help us wile away the time. He was ordered a stimulant during a portion of his treatment, and he told the doctor it was "the foinest medicin he had ever thasted in his loife," and the day it was discontinued poor Martin looked sadly crestfallen, and "would not be comforted" until, by joint representation, we convinced him that its discontinuance was a sign of convalescence.

### Yearning to Die at Home.

The man Sheldrake, a miner, before mentioned, lay week after week, patient and uncomplaining, until one day the doctor told him there was no hope, and that at any moment the "dread summons" might come. The man was anxious to go home, but knowing the utter poverty of his circumstances, the doctor humanely begged of him to stay where he was, and where he at least would be comfortable and well looked after; but the yearning to die amongst his own people, however squalid the home might be, outweighed all other considerations, and his friends themselves—almost in rags—brought a cart and took him away, and in a few days we heard that he was dead.

### A Veritable "Mark Tapley."

Another case of great hardship was that of an acrobat, a young Irishman about twenty-five years old, who, in turning somersaults on a stage, had miscalculated his distance and fallen against the wings, injuring his spine. When first he came in his injuries appeared only slight, but paralysis set in, and almost imperceptibly spread over his whole body; from day to day he could not define its progress, and it was only by weekly comparions that he was able to point to the ever-spreading numbness that must eventually snuff out the light which even yet burned so brightly in his expressive eyes; he was dying literally by inches, and he knew it, but the casual observer would never have thought so, for of all the patients Mike was the liveliest, and the sallies twixt him and Martin were a constant source of amusement. I consider Mike to have been a veritable "Mark Tapley." He was almost destitute of friends outside, for only a sister came once or twice to see him, and then she, too, fell ill, and no one came; but inside he had plenty of friends. After a time he suffered so much pain from having lain so long in one position that a water-bed was provided him. Poor fellow, his was, indeed, a pitiable case, and we cannot help feeling that his death at the moment of that fatal leap would have been more merciful. One of the prominent features of the value to a poor man of admission to a hospital is this access to the many appliances that have been invented to ease and relieve the sufferings of protracted illness—such, for instance, as poor Mike's water-bed, water-cushions, cages to keep off the pressure of bed-clothes, hot baths, armchairs on wheels, swings for broken legs, pillows innumerable for patients who cannot lie down, creosote respirators, ice-bags, feeding-cups, bed-tables, book-rests, and many other inventions, all far beyond the means of an average working man.

### A Varied Community.

Our little community was drawn from a great variety of occupations, and almost without exception from those of the working classes whose work was of a heavy laborious character, or whose occupation entailed constant exposure to cold and wet weather. Ironworkers and colliers appeared to be the most numerous, and next to those, the general labourers, the illness of many of whom could be traced to a long continued lifting of too heavy weights; men who unload a canal boat by contract, canal boatmen, agricultural labourers, carters, butchers, workers in galvanising and brass casting shops, breathing noxious fumes from morning till night; nearly all of them victims to the exacting nature of their work, a fact which the capitalist, who grows rich on their dangerous labour, while safe himself, should never lose sight of, and who would do well to come here and see these human wrecks worn down by toil in his service. Some there are, may-be, whose pitiable condition has been of their own bringing about, but I venture to say that a searching enquiry into the majority of the medical cases in a manufacturing centre would show the illness to have originated in a long continued unnatural physical strain, incumbent upon men in the earning of their daily bread on the system of piecework, for in their anxiety to make a good week, they will, like a young horse in a team, literally pull themselves to pieces. Soldiers also we had, the seeds of whose disease had been laid months before in unhealthy climates, and who had been discharged on their return to England as unfit for further service, after a few weeks sojourn at Netley; hard lines theirs after giving their best years to Queen and country. We could always pick out a soldier before he had been twenty-four hours in the ward.

## Figures and Tables

**Figure f1:**